# Evaluation of adverse effects in tamoxifen exposed healthy female dogs

**DOI:** 10.1186/1751-0147-52-67

**Published:** 2010-12-22

**Authors:** Wanessa LF Tavares, Gleidice E Lavalle, Mariana S Figueiredo, Aline G Souza, Angelica C Bertagnolli, Fernando AB Viana, Paulo RO Paes, Rubens A Carneiro, Guilherme AO Cavalcanti, Marilia M Melo, Geovanni D Cassali

**Affiliations:** 1 Laboratory of Comparative Pathology, Department of General Pathology, Institute of Biological Sciences, Federal University of Minas Gerais (UFMG), Brazil; 2 Veterinary Teaching Hospital, College of Veterinary Medicine, Federal University of Minas Gerais (UFMG), Brazil; 3 Department of Veterinary Clinical Medicine and Surgery, College of Veterinary Medicine, Federal University of Minas Gerais (UFMG), Brazil

## Abstract

**Background:**

Mammary tumors are among the most frequent neoplasms in female dogs, but the strategies employed in animal treatment are limited. In human medicine, hormone manipulation is used in cancer therapy. Tamoxifen citrate is a selective inhibitor of oestrogen receptors and exerts a potent anti-oestrogen effect on the mammary gland. The aim of this study was to evaluate the adverse effects when exposing healthy female dogs to tamoxifen.

**Methods:**

Tamoxifen was administered for 120 days at a dose of 0.5 or 0.8 mg/kg/day to either intact or spayed female dogs. The effects were assessed through clinical examination, haematology, serum biochemistry, ophthalmology and bone marrow aspirate examination. Ovariohysterectomy was performed and the uterus examined by histopathology.

**Results:**

Vulva oedema and purulent vaginal discharge developed with 10 days of tamoxifen exposure in all groups. Pyometra was diagnosed after around 90 days of exposure in intact females with frequencies increasing during the following 30 days of exposure. Up to 50% of dogs within the groups developed retinitis but none of the dogs had signs of reduced visual acuity. The prevalence of retinitis in each group was similar after 120 days of exposure. Haematological, biochemical and bone marrow changes were not observed. Due to the high risk of developing pyometra after prolonged exposure to tamoxifen, only spayed animals should be given this medication.

**Conclusions:**

A dose of 0.8 mg tamoxifen/kg body weight/day is recommended when treating tamoxifen-responsive canine mammary tumors. Due to the high risk of developing pyometra, ovariohysterectomy is recommended.

## Background

The frequency of cancer and other diseases related to aging in pet animals has increased in recent years due to increased life expectancy [1].

Mammary tumors are among the most frequent neoplasms in female dogs [1-5]. Despite their importance and high incidence, tumoral extirpation is often the preferred therapy. However, approximately 48% of dogs with mammary carcinoma die or are euthanized within one year after surgical removal of the primary tumor or recognition of metastases [6]. Thus, it is necessary to adopt low-cost alternative therapeutic approaches that can increase overall survival and welfare.

In human medicine, systemic therapies such as chemotherapy and hormone manipulation are used in cancer therapy. Tamoxifen citrate is a selective inhibitor of oestrogen receptors and exerts a potent anti-oestrogen effect on the mammary gland [7,8]. Previous studies have evaluated the efficacy of tamoxifen in preventing the recurrence of canine mammary tumors [9]. In that study, tamoxifen was administered orally at a mean dose of around 1 mg/kg body weight (BW). However, 56% of the animals developed complications such as pyometra, vulvar swelling and pseudogestational behaviour thus showing a need to assess drug tolerance in female dogs [9]. A dose of 0.5 mg/kg BW was suggested in another study to minimize adverse effects [10].

Considering the lack of non-surgical therapeutic resources for canine mammary neoplasms and the proven benefits of tamoxifen in treating human breast tumors, studies of the value of tamoxifen medication in canine medicine is needed. The epidemiological [11], clinical [4,12,13], biological [4,14], and genetic similarities [15] between human breast cancer and canine mammary tumors allow comparisons to be made [5].

The aim of this study was to evaluate adverse effects of tamoxifen exposure to healthy female dogs.

## Methods

### Animals

This study was approved by the Brazilian Committee of Ethics in Animal Experimentation, CETEA/UFMG (Protocol number 40/2006). The dogs were forwarded for adoption after completion of the study.

Healthy female mixed breed dogs aged 4 years ± 2.3 years, with a mean BW of 20 kg were used. Initially, the dogs were subjected to a clinical examination including haematology, biochemistry, ophthalmology, and bone marrow aspirate examination as described below. Dogs were only included if healthy. However, ophthalmologic changes were accepted, but dogs having eye lesions were excluded from the ophthalmology study parts.

The animals were randomly distributed into four groups:

A: 5 intact animals receiving 0.5 mg tamoxifen citrate/kg BW/day;

B: 5 spayed animals receiving 0.5 mg tamoxifen citrate/kg BW/day;

C: 5 intact animals receiving 0.8 mg tamoxifen citrate/kg BW/day;

D: 5 spayed animals receiving 0.8 mg tamoxifen citrate/kg BW/day.

Ovariohysterectomy was performed in dogs assigned to groups B and D approximately 90 days before entering the trial. The surgical procedure, anaesthetic protocol and immediate post-operative care were similar for all dogs. The animals were kept in experimental kennels from UFMG/Brazil with free access to food and water.

### Drug exposure

Tamoxifen citrate (Taxofen^® ^, Blaüsiegel, Cotia/Brazil) was administered once a day for 120 days at the same time during feeding at a dose of either 0.5 mg or 0.8 mg per kg BW. The BW was determined monthly and the total dose adjusted to the precise BW.

### Examinations

Examinations were conducted with intervals of 10 days starting with a pre-exposure examination (T00) followed by examination after 10 days of tamoxifen exposure (T01) and so on until 120 days of exposure (T02 to T12). The dogs were not euthanized at the end of the study, but ovariohysterectomy was performed at T12 for intact females (groups A and C) and the uterus subjected to pathological examination.

Clinical examination, haematology and serum biochemistry was performed at T00 to T12.

Clinical examination included evaluation of general status, measurement of rectal temperature, heart rate, and respiratory rate, inspection of mucosal membranes and skin, inspection and palpation of lymph nodes, joints, external genitalia, and mammary glands, and palpation of the abdominal cavity with contents. Abdominal ultrasonography was applied if pyometra was suspected.

Haematology was based on ethylenediaminetetraacetic acid (EDTA) stabilized blood samples taken from the cephalic vein. Serum biochemical analyses were done for alanine transaminase (ALT), aspartate transaminase (AST), gamma glutamyl transpeptidase (GGT), alkaline phosphatase (ALP), bilirubin (direct (DB), indirect (IB) and total (TB)), urea, creatinine, calcium and cholesterol.

Ophthalmological examinations were performed by a specialist at T00, T06 and T12. Two drops of mydriatic eye solution (Mydriacyl^®^, Tropicamide, 1%) were applied to each eye 15 min before examination of the fundus. Examinations were conducted with a HEINEEN 30 Indirect Ophthalmoscope and BETA 200 Direct Ophthalmoscope.

Bone marrow aspirate examinations were performed at T00, T06 and T12. The breastbone region was trimmed and disinfected with 70% ethanol. Lidocaine (2%) was used for local analgesic. Bone marrow was aspirated through a 40 × 12 hypodermic needle and a disposable 10 mL syringe. The slides were air-dried and subjected to May-Grünewald Giemsa staining before microscopy.

Samples for histopathology were fixed in 10% neutral buffered formalin, processed by routine methods for histology and embedded in paraffin. Histological sections of 4 μm were haematoxylin and eosin stained. Uterine lesions were classified as described by Dow [16].

### Statistical analysis

To evaluate the parametric data from the haemograms, biochemistry and bone marrow aspirate examination, ANOVA with a SNK test was performed. The Kruskal-Wallis test [17] was used to evaluate non-parametric haemograms, biochemical data and bone marrow aspirate results.

## Results

### Clinical findings

**Figure 1:**
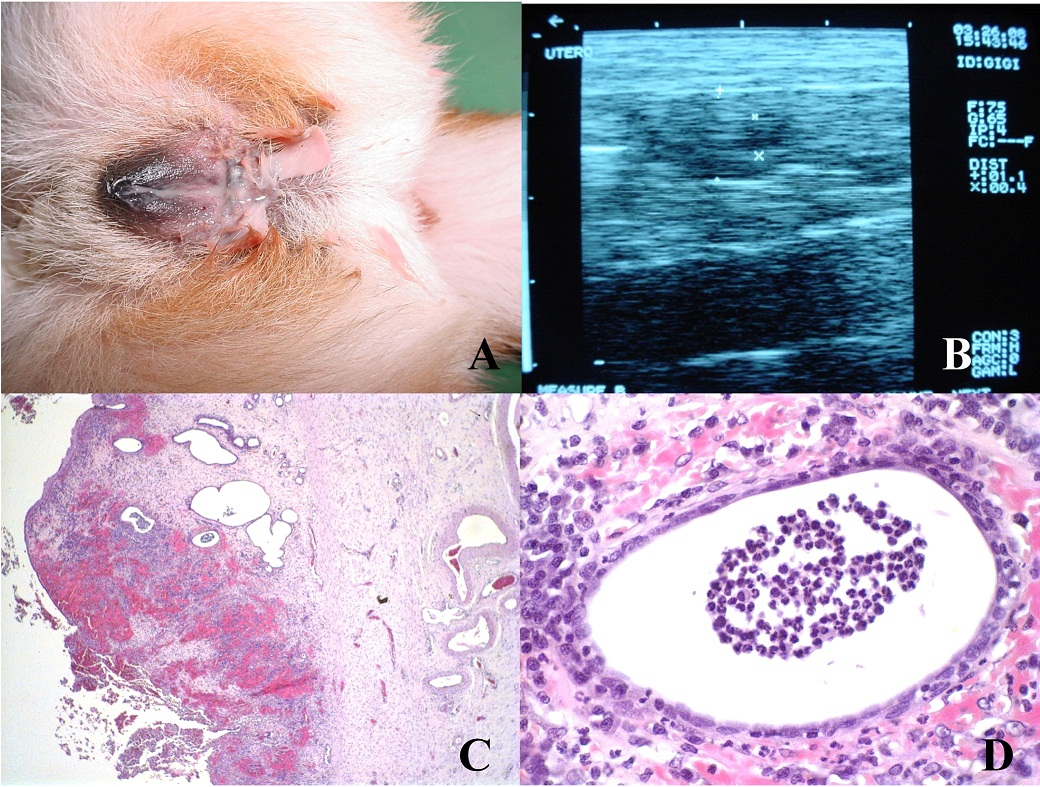
**Animal: C3, Pyometra**. (A) Intact female dog with purulent vaginal discharge after oral administration of tamoxifen, 0.8 mg/kg/day, for 100 days. (B) Ultrasonography revealing uterine body (1.5 cm) and uterine horns slightly dilated, thickened uterine wall with irregular mucosa and retention of secretion within the organ. (C) Uterine histopathology after ovariohysterectomy revealing cystic endometrial hyperplasia type III associated with hemorrhage. HE, Obj. 4×. (D) Detail showing presence of polymorphonuclear cells in glandular lumen. HE, Obj. 60×.

All dogs were found healthy before entering the trial. Vulva oedema and purulent vaginal discharge (Figure 1) developed with 10 days of tamoxifen exposure in all groups (Table 1). Pyometra (Figure 1) was diagnosed after around 90 days of exposure in intact females with frequencies increasing during the following 30 days. Two dogs developed pyometra in group A, while 4 dogs developed pyometra in group C. Rare events of vomiting, diarrhoea and appetite loss were observed throughout the period of the experiment. Dogs developing pyometra were submitted to ovariohysterectomy and excluded from the study (Table 1). Vaginal cytology was performed to evaluate oestrus cycles, but was inconclusive.

**Table 1:** Summary of genital tract symptoms and eye lesions.

		Days of tamoxyfen treatment												
**Group**	**Symptom**	**0**	**10**	**20**	**30**	**40**	**50**	**60**	**70**	**80**	**90**	**100**	**110**	**120**

A	Oedema	0	5	5	5	5	5	5	5	5	5	5	3*	3*
	Discharge	0	3	2	1	2	1	5	5	3	5	5	3*	3*
	Retinitis	1	-	-	-	-	-	1	-	-	-	-	-	3
B	Oedema	0	5	5	5	5	5	5	5	5	5	5	5	5
	Discharge	0	3	1	1	1	1	5	5	2	5	5	5	5
	Retinitis	1	-	-	-	-	-	2	-	-	-	-	-	2
C	Oedema	0	5	5	5	5	5	5	5	5	3*	3*	1^#^	1^#^
	Discharge	0	2	1	5	2	3	5	1	1	3*	3*	1^#^	1^#^
	Retinitis	1	-	-	-	-	-	1	-	-	-	-	-	1
D	Oedema	0	5	5	5	5	5	5	5	5	5	5	5	5
	Discharge	0	0	2	1	1	3	1	3	2	1	3	2	2
	Retinitis	0	-	-	-	-	-	0	-	-	-	-	-	2

* group size = 3, ^# ^group size = 1, - eyes not examined

Number of dogs having vulva oedema, purulent vaginal discharge and retinitis following oral exposure to tamoxifen at daily doses of either 0.5 mg/kg body weight (groups A and B) or 0.8 mg/kg body weight (groups C and D). Dogs in groups B and D were spayed before the study. Each group consisted initially of 5 dogs, but animals developing pyometra or had eye lesions before tamoxifen exposure were removed from the study thus reducing the group size.

### Pathology

**Figure 2:**
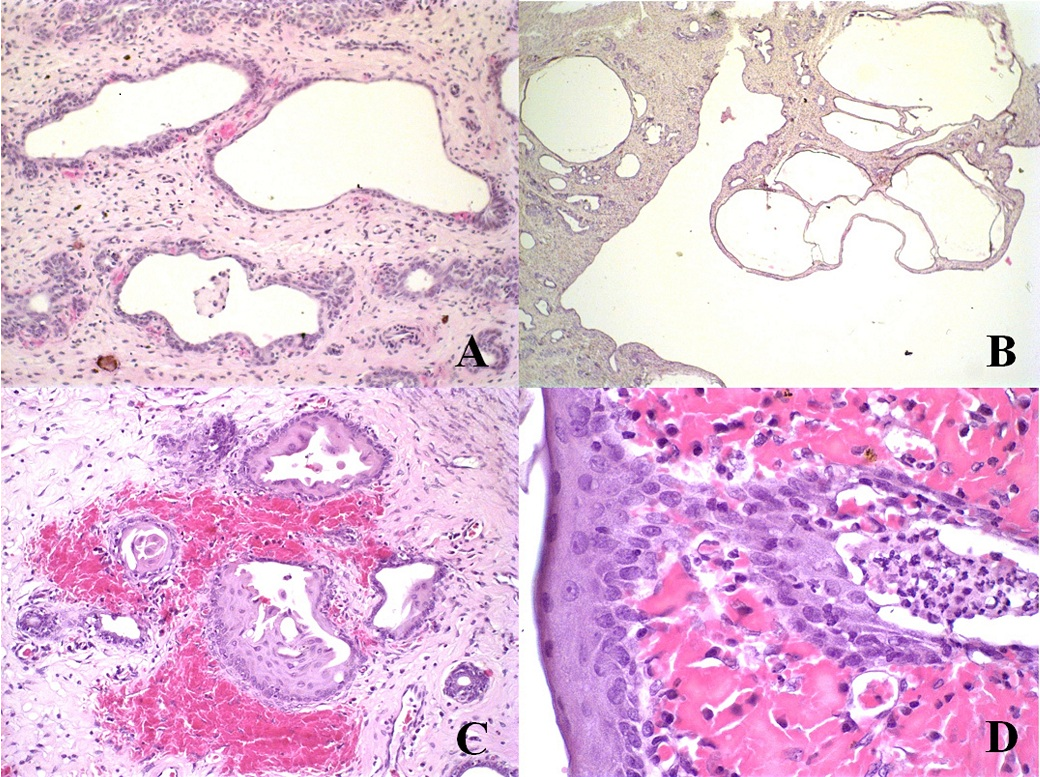
**(A and B): Animal: A2 (intact, 0.5 mg/kg/day)**. (A) Cystic endometrial hyperplasia type I. Endometrial proliferation not associated with stromal inflammatory infiltration. HE, Obj. 20×. (B) Endometrial polyp formation. HE, Obj. 4×. (C and D): Animal: A4 (intact, 0.5 mg/kg/day). (C) Cystic endometrial hyperplasia type III with squamous metaplasia of endometrial cover epithelium and hemorrhage. HE, Obj. 20×. (D) Detail showing squamous metaplasia of endometrial cover epithelium and haemorrhage. HE, Obj. 60×.

Two animals in group A developed cystic endometrial hyperplasia type I with proliferation of endometrial glands, cyst formation and endometrial polyps with no inflammatory reaction (Figure 2). The predominant pathologic findings in cases of pyometra were symmetrical distension of the uterine horns with dark-stained serosal surface and congestion. The uterine content was purulent or opaque red-brown in color. The mucosa had uneventhickness with irregular superficial haemorrhages, and in other portions obviously hyperplasia, sometimes with small cysts. Microscopically, the most significant feature was the endometrial hyperplasia associated with haemorrhage and presence of polymorphonuclear cells in the glandular lumen. The other three animals in group A presented cystic endometrial hyperplasia type III, with mononuclear and polymorphonuclear infiltration in the periglandular endometrial stroma associated with haemorrhage. Two animals in group A also presented squamous metaplasia of the endometrial epithelium (Figure 2). Only one animal in group C presented cystic endometrial hyperplasia type I, while three in this group developed cystic endometrial hyperplasia type II associated with mononuclear cells (macrophages, lymphocytes and plasmacells) with superficial subepithelial haemorrhage. One animal also developed squamous metaplasia and endometrial polyps. One animal presented cystic endometrial hyperplasia type III (Figure 1).

### Ophthalmology

Three animals (A4, B3 and C5) that had ophthalmological alterations at T00 were excluded. Up to 50% of dogs within the groups developed retinitis during the study (Table 1). Once a dog had developed retinitis, this condition remained throughout the study. Retinitis was characterised by the presence of black dots on tapetal area [18]. None of the dogs had signs of reduced visual acuity. The prevalence of retinitis at T12 in the exposure groups (A+B: 0.5 mg and C+D: 0.8 mg) was non-significant.

### Haematology and biochemistry

Evaluation of haematological and biochemical profiles were restricted to the period T00 to T08 as several dogs were removed from the study during the last time of the trial (Table 1). The mean values for erythrocytes, haemoglobin, globular volume, platelets, ALT, AST, GGT, ALP, IB and calcium remained within the normal ranges for all dogs [19].

Slight increases were observed in the mean values of total leukocytes at T01, T02 and T07 in group B and T02 in group D (data not shown).

### Bone marrow aspirate examination

The mean values of cellularity, megakaryocytes, metarubricytes, lymphocytes, plasmacells and monocytes remained within the normal range [20] for all dogs throughout the trial.

## Discussion

Oedema of the external genitalia as seen in dogs of all groups develops due to the agonist action of tamoxifen on genital hormonal receptors. After ovariohysterectomy, the uterine stump may develop pyometra. This was not observed in the present study (groups B and D) although ultrasonography was applied to all animals having vaginal discharge. The cause of vaginal discharge in these animals remained unsolved.

The agonist action of tamoxifen on the human uterus promotes adverse effects such as endometritis and endometrial hyperplasia. These are considered pre-malignant lesions thus increasing the risk for development of endometrial carcinoma [21-24]. It is evident that, as in women, intact female dogs exposed to tamoxifen develop endometrial cell proliferation most likely due to the agonist stimulation of uterine oestrogen receptors. The results corroborate previous studies [9,10] suggesting that tamoxifen causes oestrogenic stimulation of canine endometrial cells even in low doses. The number of oestrogen receptors increases due to hormonal stimulation with consequent endometrial hyperplasia and an increased number of progesterone receptors [9]. The serum level of this hormone remains unaltered, but as the number of receptors is increased, leukocyte recruitment to the uterus is reduced and uterine shrinkage is impaired. This process promotes reduction of uterine immune defences and facilitates ascending bacterial infections, mainly caused by *Escherichia coli*, unleashing pyometra [25].

Three animals from groups A and C presented squamous metaplasia of the endometrial epithelium, probably due to estrogenic stimulation as seen in women treated with tamoxifen [26,27] and dogs with spontaneous cystic endometrial hyperplasia complex-pyometra [28]. Endometrial polyps were found in one case. This type of lesion has also been reported in women after treatment with tamoxifen [21,22,26].

The ophthalmological alterations are similar to those found in women treated with tamoxifen at 20 mg dose per kg BW for 5 years. However, the prevalence seems to be higher in dogs even though they were exposed to a lower dose and for a shorter period. This discrepancy may be related to a difference in species sensibility, since the mechanisms of action of tamoxifen are related to specific variant oestrogen receptor expression in different cell types and different mechanisms of DNA-receptor interaction. Thus, tamoxifen acts more as agonist than antagonist in canines thus suggesting species differences [29]. The lower dose groups (A and B) presented ophthalmological lesions after 60 days of exposure suggesting variation in sensibility not related to dose or hormone levels (intact or spayed animal). In human medicine, eye lesions are reversible after suspension or termination of treatment [30-33]. It is therefore expected that such lesions will also be reversible in canine species after treatment is suspended.

On the basis of previous studies [9,10], doses of 0.5 and 0.8 mg/kg BW/day were chosen. Considering the risk of developing pyometra when administering tamoxifen to intact female dogs, it is suggested that this medication be prescribed to spayed animals only. Since there was no difference between the lower and higher dose groups in the other side-effects induced by tamoxifen, the higher dose may increase the chances of therapeutic success.

## Conclusions

Tamoxifen may become an important compound in veterinary medicine considering its therapeutic potential for increasing the overall survival rate of female dogs with mammary tumors, if its side effects are correctly assessed and controlled. A dose of 0.8 mg tamoxifen/kg BW/day for at least 120 days is recommended.

## Competing interests

The authors declare that they have no competing interests.

## Authors' contributions

WLFT was responsible for all procedures and drafted the manuscript. GEL performed surgery and participated in the study design. MSF and AGS participated in kennel maintenance, drug administration, sample collections and surgical procedures. ACB conducted vaginal cytology. FABV was the ophthalmological specialist responsible for eye examinations. PROP performed myelogram examinations, whose samples were collected by RAC. GAOC conduced ultrasonography examinations. MMM participated in the study design and was the co-advisor of this work. GDC was the advisor of this work. All authors read and approved the final manuscript.
